# Advanced Robotics for the Next-Generation of Cardiac Interventions

**DOI:** 10.3390/mi16040363

**Published:** 2025-03-22

**Authors:** Majid Roshanfar, Mohammadhossein Salimi, Amir Hossein Kaboodrangi, Sun-Joo Jang, Albert J. Sinusas, Shing-Chiu Wong, Bobak Mosadegh

**Affiliations:** 1 Department of Mechanical Engineering, Gina Cody School of Engineering, Concordia University, Montreal, QC H3G 1M8, Canada; m_roshan@encs.concordia.ca; 2 Department of Mechanical Engineering, York University, Toronto, ON M3J 1P3, Canada; mhsalimi@yorku.ca; 3 Department of Surgery, Broward Health, Fort Lauderdale, FL 33316, USA; akaboodrangi@browardhealth.org; 4 Section of Cardiovascular Medicine, Department of Medicine, Yale University School of Medicine, New Haven, CT 06519, USA; sun-joo.jang@yale.edu (S.-J.J.); albert.sinusas@yale.edu (A.J.S.); 5 Division of Cardiology, Department of Medicine, Weill Cornell Medicine, New York, NY 10021, USA; scwong@med.cornell.edu; 6 Dalio Institute of Cardiovascular Imaging, Department of Radiology, Weill Cornell Medicine, New York, NY 10021, USA

**Keywords:** artificial intelligence, autonomous navigation, cardiac intervention, haptic feedback, soft robotics, telerobotics

## Abstract

With an increasing number of elderly individuals, the demand for advanced technologies to treat cardiac diseases has become more critical than ever. Additionally, there is a pressing need to reduce the learning curve for cardiac interventionalists to keep pace with the rapid development of new types of procedures and devices and to expand the adoption of established procedures in more hospitals. This comprehensive review aims to shed light on recent advancements in novel robotic systems for cardiac interventions. To do so, this review provides a brief overview of the history of previously developed robotic systems and describes the necessity for advanced technologies for cardiac interventions to address the technological limitations of current systems. Moreover, this review explores the potential of cutting-edge technologies and methods in developing the next generation of intra-procedure autonomous navigation. Each highlighted topic undergoes a critical analysis to evaluate its technical limitations and the challenges that must be addressed for successful clinical implementation.

## 1. Introduction

### 1.1. Robot-Assisted Interventions: System Overview

The field of interventional cardiology has experienced significant progress over recent decades, with percutaneous coronary intervention (PCI) becoming the preferred approach for patients who have coronary artery disease (CAD) [1,2]. Despite the benefits, these procedures expose interventional cardiologists to occupational risks, particularly radiation [3]. Increasing awareness of these risks has driven the advancement of robotic systems for cardiovascular interventions [4,5]. Furthermore, robotic systems offer enhanced precision, enabling submillimeter movements and acceptable rotations, which can be difficult to achieve manually [6,7]. Recent advances have further demonstrated the exceptional precision capabilities of these systems in achieving consistent submillimeter accuracy for complex cardiac procedures [8,9].

Medical robotics for cardiovascular applications began to develop in the late 1980s and 1990s, driven by the desire to enhance surgeon capabilities and overcome human limitations. Early developments included robotic systems for cardiac surgery, such as the ROBODOC system (1992) for orthopedic applications, and early prototypes that would eventually evolve into the da Vinci Surgical System. These pioneering systems demonstrated the potential benefits of robotic assistance in terms of precision and reduced invasiveness, but focused primarily on open or laparoscopic surgical approaches rather than endovascular interventions. The transition to catheter-based robotic systems came in the late 1990s with research prototypes exploring remote manipulation of catheters and guidewires, though these systems were limited in their degrees of freedom and lacked the sophisticated control interfaces seen in later commercial systems. These efforts culminated in the early 2000s, with the first robotic electrophysiological procedure performed by Ernst et al. [10]. This work eventually led to Hansen Medical’s development of the Sensei Robotics Catheter System in 2004, marking the first robotic catheter system for endovascular use. Shortly after, robotic PCI emerged, with Beyar et al. conducting the first robotic PCI on human subjects in 2006 [11]. Their system included an operator module with a touchscreen and joystick and a patient module containing a robot with three degrees of freedom (DoFs) to manipulate guidewires and devices. The first commercially available robotic PCI system, CorPath 200 by Corindus Inc., was launched in 2012. It evolved from earlier remote navigation systems, like the one used by Beyar et al., and initially received FDA approval for PCI procedures.

**Figure 1:**
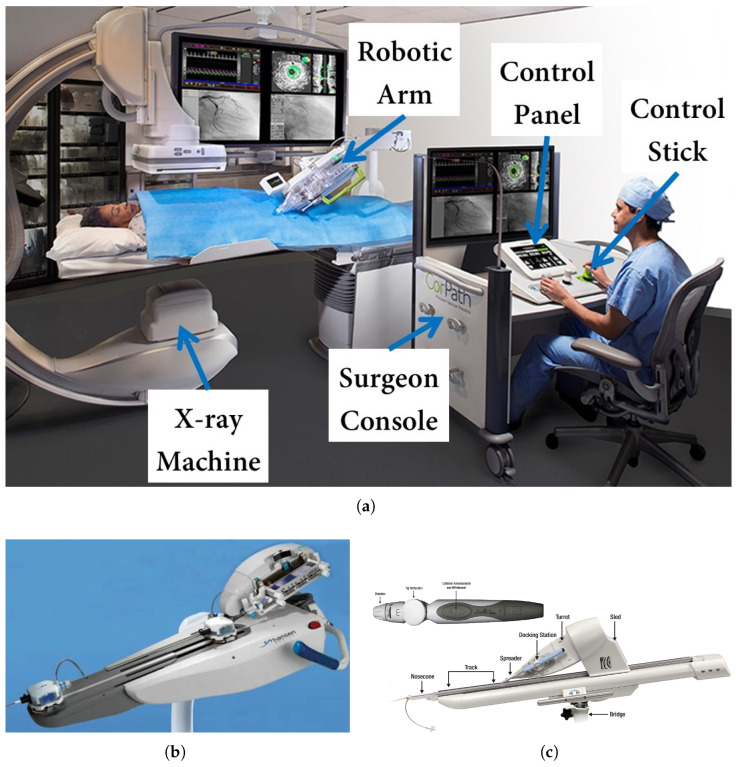
FDA-approved robot-assisted systems for cardiovascular interventions. Images reproduced with permission. (**a**) CorPath GRX System including control console and robotic driver (courtesy of Corindus Inc., Waltham, MA, USA), (**b**) Magellan Vascular Robotic System (courtesy of Hansen Medical, Mountain View, CA, USA) [13], (**c**) Amigo Remote Catheter System with handheld controller (courtesy of Catheter Precision, Inc., Mount Olive, NJ, USA) [15].

Today, several FDA-approved robotic systems are in use for cardiovascular interventions. The CorPath GRX System (Corindus/Siemens Healthineers) was the most widely used, and it gained FDA clearance for CI in 2012 and peripheral interventions (PIs) in 2018 [12]. The system consists of a control station for the operator, a robotic arm beside the patient, and a sterile disposable cassette for device manipulation. It controls guidewires, stents, and balloons precisely, enabling 1 mm incremental movements and 30-degree rotations. Next, Hansen Medical introduced the Magellan Vascular Robotic System in 2013, explicitly designed for peripheral vascular interventions (PVIs) [13]. One key difference between the CorPath and Magellan systems is catheter compatibility. The CorPath systems accommodate most commercial cardiac catheters, while the Magellan system works exclusively with proprietary steerable catheters. Its steerable catheters, operated by a cable-driven mechanism, can bend at two perpendicular axes, allowing for distal tip control during insertion. This increased maneuverability improves navigation through complex vascular structures, a feature unavailable in systems like CorPath, which rely on proximal control. In addition, the FDA approved the Amigo Remote Catheter System (Catheter Precision, Inc., Mount Olive, NJ, USA) for electrophysiology in 2012 [14]. This system is designed for use in cardiac ablation procedures and allows operators to manipulate catheters remotely and outside the radiation field. A key feature of the Amigo system is its ability to reproduce linear catheter motions, rotary motion, and tip deflection. These movements are controlled through appropriate buttons on the handheld remote controller, allowing for intuitive, one-handed operation. This design aims to mimic the natural hand movements of the interventionalist, potentially reducing the learning curve associated with adopting robotic technology [15,16]. Figure 1 shows the CorPath, Magellan, and Amigo robotic systems for cardiovascular interventions.

### 1.2. Necessity for Advanced Technologies for Cardiac Intervention

Integrating advanced technologies into cardiac intervention procedures has become increasingly necessary due to several critical factors. A primary driver is the need to mitigate occupational hazards faced by interventionalists, including radiation exposure and the physical tolls exerted on operators in increasingly complex procedures. Additionally, studies have reported that complications arising from imprecise stenosis localization, inaccurate lesion length estimation, and stent misplacement are among the most common risks for patients undergoing PCI [17]. These challenges can result in suboptimal outcomes and often necessitate repeat procedures. Also, the lack of haptic feedback is a critical limitation of most robotic systems for cardiac intervention [18]. Although robotic platforms have effectively reduced operator radiation exposure [19], the absence of tactile sensation, which surgeons rely on in conventional procedures, remains a significant barrier to broader adoption [20]. A new type of actuation system also needs to be developed and integrated to overcome the limited steerability issues present in some current robotic systems.

Moreover, advancements in imaging technologies for navigation have been identified as vital for increasing the precision and safety of cardiac interventions. Traditional fluoroscopy, while offering real-time imaging with a large field of view, lacks the 3D spatial information (i.e., depth perception) critical for complex procedures. Studies demonstrate that integrating 3D imaging modalities, such as computed tomography (CT) angiography, with real-time 2D fluoroscopy can significantly improve navigation and procedural outcomes [21]. The fusion of imaging technologies enables more intuitive visualization of vascular anatomy and more precise tracking of interventional devices. Moreover, using artificial intelligence (AI) with X-ray visualization is a promising step toward technological advancements in fully autonomous robotic systems. AI-powered image processing algorithms have the potential to reduce radiation dose while maintaining or improving image quality [22]. These technologies help automatically identify anatomical structures, enhance vasculature visibility, and assist in real-time decision-making during procedures. However, real-time data fusion in robotic navigation presents challenges, particularly in managing image registration errors caused by patient motion, soft tissue deformation, and variations in imaging modalities. To mitigate these issues, advanced image registration techniques such as AI-driven deformable models, fiducial marker-based alignment, and real-time sensor fusion methods (e.g., electromagnetic tracking combined with fluoroscopy) are employed. Continuous recalibration and adaptive filtering techniques help maintain registration accuracy, ensuring precise robotic guidance throughout the procedure.

### 1.3. Literature Survey

This review explores advanced robotic technologies for the next generation of cardiac interventions, focusing on recent advancements in robotic systems, AI-driven navigation, and soft robotic applications. The literature search was conducted to identify relevant studies published from 2014 onward using databases such as PubMed, IEEE Xplore, Web of Science, Compendex, and Scopus. The selection of papers was guided by their relevance to robotic-assisted cardiac interventions, AI-based navigation, soft robotics for endovascular applications, and haptic feedback technologies. Particular attention was given to studies introducing novel robotic platforms, innovative control strategies, and advancements in surgical automation.

To ensure that this review covered impactful contributions, preference was given to papers that demonstrated experimental validation, preclinical studies, or clinical applications, highlighting their practical significance. Only peer-reviewed journal articles and conference proceedings from reputable publishers were considered. Papers were included based on their alignment with the key themes of this review and their contribution to understanding the role of robotics in cardiac interventions. A broad initial pool of studies was identified, and non-relevant papers were excluded based on their abstracts and conclusions. The final selection consisted of 42 papers that provided significant insights into the technological landscape of robotic-assisted cardiac interventions. Unlike previous reviews that primarily focused on early robotic systems or specific clinical applications, this paper provides a comprehensive perspective on the latest technological breakthroughs, particularly in AI-assisted navigation, soft robotics, and multimodal imaging integration. By addressing existing system limitations such as the lack of real-time adaptability, haptic feedback, and precision challenges, this review highlights emerging solutions that set the foundation for the next generation of cardiac robotic interventions.

### 1.4. Motivations for Next-Generation Systems

**Figure 2:**
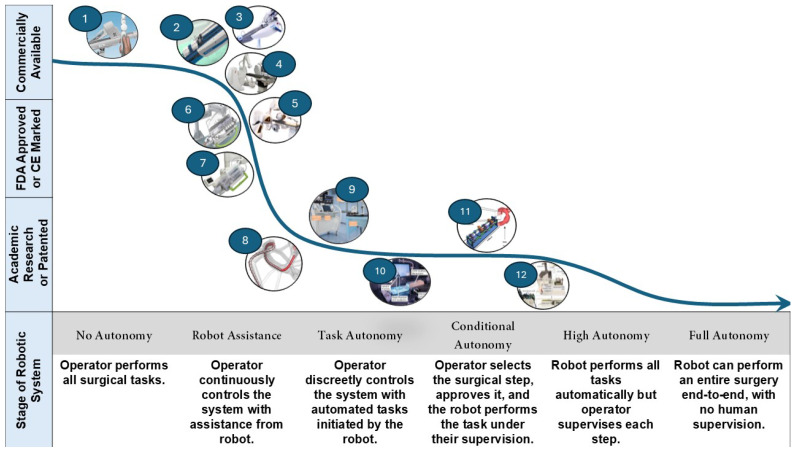
Classes of robot autonomy in cardiac intervention. (1) CRI Amigo System, Catheter Precision, Inc., Mount Olive, NJ, USA. (2) Sensei Robotics Catheter System, Hansen Medical, Mountain View, CA, USA. (3) Magellan Robotic Catheter System, Hansen Medical, Mountain View, CA, USA. (4) Niobe Remote Magnetic Navigation System, Stereotaxis, St. Louis, Mo, USA. (5) Genesis Remote Magnetic Navigation System, Stereotaxis, St. Louis, Mo, USA. (6) CorPath 200, Corindus Inc., Waltham, MA, USA. (7) CorPath GRX System, Corindus Inc., Waltham, MA, USA. (8) Magnetically actuated robot by Dreyfus R. et al. [28]. (9) Wang S. et al. [29]. (10) Karstensen L. et al. [30]. (11) Jianu T. et al. [31]. (12) Wang Y. et al. [32]. Images reproduced with permission.

The progression toward autonomy in robot-assisted cardiac interventions represents a significant focus of current research efforts [23,24]. As illustrated in Figure 2, there is a trajectory from operator-controlled systems with robotic assistance to increasingly autonomous operations [25]. This evolution begins with basic robotic assistance, where the operator maintains continuous control and advances through stages of task autonomy and conditional autonomy. In these intermediate stages, the robot can initiate automated tasks or perform specific steps under operator supervision. The ultimate goal is to achieve high or full autonomy, where robotic systems can perform entire surgeries end-to-end with minimal or no human supervision. This push towards autonomy is motivated by several key factors. Firstly, it has the potential to improve clinical outcomes by enhancing precision and consistency in surgical procedures. Robotic systems can operate with a level of steadiness and accuracy that surpasses human capabilities, particularly in delicate cardiac procedures. Secondly, increased autonomy could help reduce human error, which remains a concern in complex surgical interventions [26]. Automating certain aspects of the procedure can minimize the risk of mistakes. However, the journey towards truly autonomous cardiac interventions is not without challenges. As these technologies advance, addressing regulatory hurdles becomes increasingly crucial [27].

Ensuring patient safety with highly autonomous systems requires rigorous testing and validation protocols. There are also ethical considerations to navigate, such as determining the appropriate level of human oversight and establishing clear lines of responsibility in autonomous procedures. Another critical challenge in adopting robotic-assisted cardiac interventions is the steep learning curve associated with current systems, which require extensive operator training to achieve proficiency. Traditional robotic platforms demand manual control with minimal automation, necessitating specialized training programs. However, emerging technologies, such as AI-assisted navigation and enhanced haptic feedback, are reducing cognitive workload and improving ease of use. AI-driven systems provide real-time guidance and semi-autonomous control, while advanced haptic interfaces restore tactile sensation, making robotic procedures more intuitive. Studies have shown that these advancements contribute to shorter training times and improved procedural efficiency, enhancing overall usability and clinical adoption. In this review, however, we will focus on the technical advancements and novel robotic technologies that have been developed in the last decade.

## 2. Advanced Robotics in Cardiac Interventions

### 2.1. Robot-Assisted Interventions: Clinical Outcomes and Limitations

Robot-assisted cardiac intervention has been shown to be safe and feasible. Clinical studies like the PRECISE trial [33] and CORA-PCI study [34] have demonstrated high clinical and technical success rates with robotic PCI comparable to manual PCI. The PRECISE trial reported a clinical success rate of 97.6% and technical success of 98.8% in 164 patients with mostly simple lesions. The CORA-PCI study, which included a higher proportion of complex lesions, showed a clinical success of 99.1% and technical success of 91.7%. Robotic PCI has been associated with significant reductions in operator radiation exposure of 95–97% compared to manual PCI [35]. Studies have also shown the feasibility of robotic PCI via both radial and femoral access routes. However, some limitations of current robotic PCI systems have been identified. The lack of active guide catheter control in first-generation systems like the CorPath 200 was a common reason for manual assistance or conversion during procedures. Newer systems like the CorPath GRX have incorporated active guide control to address this.

However, the lack of haptic feedback and limited stability could be added to the list of limitations that hindered the better outcomes of such robotic systems. To overcome these limitations, several promising approaches could be implemented in future systems. For haptic feedback challenges, integrating multimodal feedback combining visual, auditory, and tactile cues could enhance operator perception without requiring complex mechanical systems. Advanced machine learning (ML) algorithms could also translate imaging data into simulated force feedback, creating an augmented sensory experience. For steerability limitations, hybrid actuation mechanisms combining traditional cable-driven systems with magnetic [36] or pneumatic soft actuators [37,38,39,40] could provide enhanced flexibility while maintaining precise control. Another approach involves implementing variable stiffness mechanisms in catheter designs, allowing operators to selectively increase rigidity for navigation through challenging anatomy while maintaining softness for delicate tissue interactions [41]. Additionally, implementing shared control paradigms where autonomous features complement manual operation could help overcome both limitations simultaneously, as the system could provide haptic cues based on pre-programmed constraints while enhancing navigation capabilities through partial automation of repetitive tasks. These innovative solutions align with the ongoing technological advancements discussed in the following sections, particularly in the areas of control accuracy and position control.

#### 2.1.1. Control Accuracy

**Figure 3:**
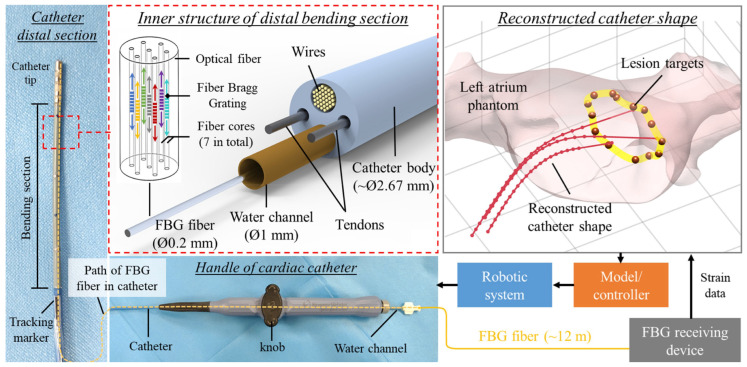
FBG-based shape-sensing architecture used inside the inner channel of the ablation catheter [43]. Image reproduced with permission.

Control accuracy is a critical aspect of robotic systems for cardiac interventions, where precise manipulation is essential for procedural efficiency and minimizing risks. Wang et al. developed a cardiovascular interventional surgery robot with an integrated force sensor and proposed a novel force/position controller combining a radial basis function neural network (NN) position controller and an admittance controller [42]. They conducted simulations and vascular model experiments to evaluate the controller’s performance in improving guidewire delivery accuracy and reducing contact force with vessel walls. The results demonstrated that the proposed controller enhanced guidewire delivery precision and decreased the maximum contact force from 0.96 N to 0.34 N in one specific vessel branch. Similarly, Dong et al. explored control accuracy in cardiac interventions by developing a shape tracking and feedback control system for cardiac catheters during magnetic resonance imaging (MRI)-guided electrophysiology procedures [43]. They integrated a multi-core fiber Bragg grating (FBG) sensor [44] and magnetic resonance (MR) tracking coils into a standard catheter, enabling real-time 3D shape reconstruction and position sensing. They proposed a learning-based piecewise constant curvature model to characterize the catheter kinematics and implemented it on an MR-safe robotic platform for autonomous control. Experimental results demonstrated accurate shape sensing (2.33° average error) and precise tip positioning (1.53 mm accuracy). They showcased the potential of combining fiber optic shape sensing with MR-based tracking for improved catheter navigation and control under MRI guidance. As illustrated in Figure 3, Dong et al.’s approach addressed the challenges of modeling uncertainties in commercial catheters and provided real-time shape feedback for visualization and robotic manipulation.

#### 2.1.2. Haptic Feedback and Haptic Devices

Recent advancements in haptic feedback systems have shown potential for restoring tactile sensation in robotic-assisted minimally invasive surgery (MIS) surgical procedures [45]. Various soft sensing technologies [46], electrical impedance tomography, and sensorless methods [47,48] have been developed to enhance the capabilities of robotic systems. The effectiveness of haptic feedback systems in cardiac interventional robotics is typically evaluated using several quantitative and qualitative metrics. Quantitative metrics include: (1) force discrimination threshold (the minimum detectable force difference, typically measured in newtons), (2) position accuracy (measured in millimeters or degrees for rotational movements), (3) task completion time, (4) success rate of target acquisition, and (5) learning curve reduction. Qualitative assessments often involve user experience questionnaires measuring cognitive workload, user comfort, and perceived usefulness. For example, in the study by Hooshiar et al. [49], the magnetostriction-based force feedback system was evaluated based on its ability to generate resistant torques (up to 115.5 mN.m) and how these compared to the minimum required feedback for accurate perception during procedures. Similarly, Tahir et al. [50] measured task completion time reduction (37%) and operator performance with different feedback modalities when compared to no feedback conditions. These standardized metrics enable objective comparison between different haptic feedback approaches and highlight the direct correlation between effective haptic feedback and improved procedural outcomes. Building upon these evaluation frameworks, recent advancements in haptic feedback methods have emerged to improve user interaction and control precision.

**Figure 4:**
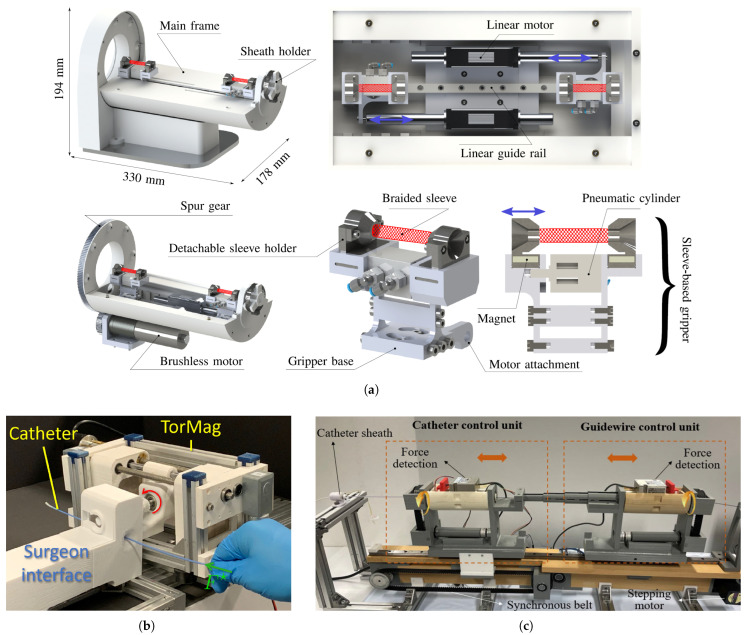
Haptic feedback and haptic devices for cardiovascular interventions. Images reproduced with permission. (**a**) Isometric and sectional views of RACS, consisting of a braided sleeve that is pneumatically actuated [52], (**b**) TorMag’s haptic device in the surgeon interface, which provides friction torque on the shaft of the catheter by deforming the MRE material [49], (**c**) Manipulator integrated with operating units and a haptic interface [53].

Alongside these advancements, haptic feedback methods, including pneumatic actuators and vibrotactile systems [51], have emerged to improve user interaction and control precision. Al-Ahmad et al. developed a novel robot-assisted catheterization system using braided sleeve grippers, enabling continuous instrument motion and precise control [52]. They implemented a force control strategy with hysteresis compensation and utilized FBG-inscribed multi-core fibers for real-time instrument tip pose tracking. The system demonstrated superior force tracking performance and was successfully validated in in vivo experiments on a live swine. Li et al. developed a dual-instrument vascular interventional system with a novel soft magnetic-actuation-based haptic interface for robot-assisted surgery [53]. They designed a safety operation strategy and conducted experiments to evaluate the system’s feasibility, tracking performance, and operating performance. The results showed a maximum linear error of 1.87 mm, haptic feedback range of 0.01–1.49 N, and improved safety during operations. Bao et al. developed a novel haptic interface for robot-assisted endovascular catheterization that retains tactile sensation and traditional operating modes while providing force and torque feedback [54]. They evaluated the device’s performance through simulations, laboratory experiments, and animal trials comparing it to commercial haptic interfaces. The results showed the proposed interface had better accuracy, lower completion times, and reduced operator workload compared to existing systems, particularly for complex tasks. Figure 4 illustrates the aforementioned haptic interfaces designed for robot-assisted cardiac intervention.

These haptic interfaces present important trade-offs for clinical implementation. The braided sleeve gripper system offers exceptional force tracking precision but requires specialized catheters, potentially increasing costs and limiting compatibility with existing tools. The magnetic-actuation-based system provides a clinically relevant haptic feedback range (0.01–1.49 N) with reasonable positioning accuracy, though implementing safe magnetic systems introduces additional complexity. Bao’s interface excels in reducing operator workload and improving task completion times, particularly beneficial for complex anatomies, but may require significant training investment. From a clinical feasibility perspective, TorMag’s magnetostriction approach facilitates easier sterilization and integration into catheterization laboratories by avoiding mechanical coupling, while hydraulic-based systems offer superior force capabilities but introduce maintenance and reliability considerations. Future clinical adoption will likely depend on balancing these trade-offs against specific procedural requirements, with optimal solutions potentially incorporating elements from multiple approaches based on intervention complexity and healthcare economic factors.

#### 2.1.3. Position Control

Jolaei et al. developed a learning-based control framework for kinematic control of flexible ablation catheters, aiming to achieve task autonomy in robotic cardiac ablation [55]. They utilized support vector machine classification for tendon selection and NN regression for tendon length estimation, implementing the control system on a custom-built robotic catheter intervention platform. The proposed method demonstrated submillimeter accuracy in trajectory tracking and target-reaching tasks, with average root-mean-square errors of 0.49 mm and 0.62 mm for slow and fast trajectories, respectively. Kesner et al. developed a robotic catheter system capable of compensating for the rapid motion of cardiac structures during beating heart surgery, using 3D ultrasound imaging for guidance [56]. They identified friction and backlash as major limitations in catheter control, and implemented mechanical design improvements and control methods, including inverse and model-based backlash compensation, to mitigate these issues. In vivo experiments demonstrated that the system could track mitral valve motion with root-mean-square errors of less than 1 mm. While this work shows promise for enabling new minimally invasive intracardiac procedures, further developments in multi-DoF actuation, force sensing, and specialized end-effectors are needed to expand the range of possible interventions.

**Figure 5:**
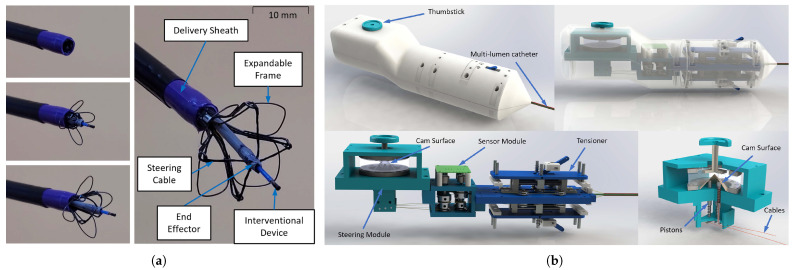
CathPilot for control of flexible ablation catheters [57]. (**a**) Catheter lumen and expandable frame, (**b**) Internal mechanisms of the user input handle unit. Images reproduced with permission.

Zhou et al. developed the CathPilot, a novel expandable cable-driven parallel manipulator for accurate interventional device steering and tracking in MI cardiovascular procedures [57]. The system utilizes an expandable frame, four cables, and a 3D cam surface for cable length adjustment, allowing for teleoperation and submillimeter-accurate positioning within the frame’s workspace. Experimental results demonstrated impressive accuracy, with mean tracking errors below 2.2% of the workspace size (10–20 mm diameter) across various configurations and expansion sizes. The CathPilot maintained submillimeter positioning accuracy regardless of path tortuosity and catheter shape, while tracking errors for untrained intermediate frame sizes ranged from 3.34% to 5.01%. The CathPilot significantly outperformed conventional catheters in phantom lesion crossing tests, achieving significantly faster crossing times and a 100% success rate compared to occasional failures with conventional methods. The main advantages of this approach include navigating complex anatomy, real-time 3D tracking with X-ray fluoroscopy, and intuitive control with haptic feedback. However, potential limitations include assumptions about frame symmetry and the need for further miniaturization. Figure 5 illustrates the overview of CathPilot for the control of flexible ablation catheters.

#### 2.1.4. Catheter Tracking and Autonomous Navigation

Accurate catheter tracking is essential for precise navigation and successful outcomes in cardiac interventional procedures [58,59,60,61]. A recent study introduces an innovative extended reality (XR) platform designed for real-time 3D catheter tracking and visualization during simulated cardiac interventions. The system leverages a custom 3D-printed setup with two orthogonally positioned cameras that capture a biplane video of catheter movements. Real-time processing of these biplane images through an advanced computer vision algorithm enables 3D trajectory reconstruction with high precision, achieving tip localization accuracy within 1 mm. The tracking data are integrated into a Unity-based visualization system and displayed on a Meta Quest 3 headset, allowing the catheter’s position to be visualized within a patient-specific 3D heart model reconstructed from CT scans. Validation experiments using commercial catheters and 3D-printed phantoms demonstrated highly accurate shape reconstruction with minimal angular errors. A user study involving six participants highlighted the advantages of 3D visualization, with users completing tasks about 7.3 times faster compared to traditional 2D visualization methods [62].

Chang et al. present a robust method for tracking catheters in fluoroscopic images [63]. Their main contribution is a novel B-spline tube model combined with a probabilistic framework that uses pixel-wise posteriors for fitting and tracking. The approach can effectively represent complex catheter shapes and handle challenges like low signal-to-noise ratio, thin instrument appearance, and overlapping segments. The method uses a knot-driven B-spline representation for better local control and optimizes the contour with respect to knot points, incorporating an equidistance prior to avoid collisions. They evaluate their approach on both phantom and clinical data, demonstrating high accuracy with an average missing rate of less than 4% pixels over the entire tracked catheter length. However, limitations include potential difficulties in tracking when guidewire measurements appear fragmentary or when there is rapid motion causing blur or instrument disappearance. The semi-automatic nature of the system, requiring some user input for initialization and knot point management, could also be seen as a limitation for fully automated applications. Chi et al. present a learning-based robotic catheterization platform that optimizes endovascular navigation trajectories using reinforcement learning (RL) [64]. They combine dynamic movement primitives learned from expert demonstrations with policy improvement through path integral RL to generate optimized catheter motion trajectories. The system aims to reduce unwanted contacts between the catheter tip and vessel walls during cannulation tasks in aortic arch phantoms. They evaluate their approach in standard, aneurysm, and stenosis Type I aortic arch models under dry, continuous flow, and pulsatile flow conditions. Results show the optimized robotic approach achieves significantly shorter catheter path lengths, lower root-mean-square errors to vessel centerlines, and reduced contact forces compared to manual catheterization and non-optimized robotic approaches. Key advantages include improved safety through reduced vessel contacts and forces. However, current AI-driven navigation approaches still face challenges such as longer procedure times, reliance on preoperative 3D models, and the potential risks associated with model biases and overfitting.

One of the primary concerns in RL and AI-driven trajectory optimization is ensuring model robustness and generalizability while minimizing biases in training data. Various strategies have been explored to mitigate these risks. Domain randomization techniques introduce variations in anatomical structures, imaging conditions, and catheter–tissue interactions, helping models adapt to diverse patient anatomies. Additionally, incorporating extensive and diverse datasets, including real-world and simulated patient-specific scenarios, enhances generalization. Regularization methods, such as dropout and L2 normalization in NN architectures, help prevent overfitting, while transfer learning approaches allow models to be pre-trained on large-scale synthetic datasets before fine-tuning on limited real-world data. Rigorous validation in both simulated and experimental environments remains crucial to assessing model reliability before clinical translation. These strategies collectively contribute to improving the adaptability and safety of AI-driven catheter navigation systems.

Current autonomous navigation systems demonstrate varied success rates depending on procedural complexity: high rates in controlled settings (97–98% for basic navigation [65]) and promising results in animal studies (95% success for intracardiac navigation [66]), but significant decline for complex tasks like dual device manipulation (40% success [65]). Also, handling patient-specific anatomical variations and dynamic cardiac motion remains a challenge in AI-driven catheter navigation. Preoperative imaging data (CT/MRI) can be used to tailor navigation strategies, while real-time sensor fusion (e.g., fluoroscopy, intracardiac echocardiography) helps adjust trajectories dynamically. ML models trained with domain randomization improve adaptability to anatomical differences, and predictive modeling techniques, such as Kalman filters, help compensate for cardiac motion. Several barriers challenge clinical integration, including regulatory hurdles requiring extensive validation, cost-effectiveness concerns given high capital investment, workflow disruption, learning curves for clinical teams, infrastructure limitations in catheterization laboratories, and unresolved liability questions regarding autonomy levels and responsibility allocation. Despite these challenges, emerging approaches such as the Wang system [32] demonstrate encouraging progress toward the clinical feasibility of autonomous navigation in cardiac interventions. In addition, Fagogenis et al. demonstrated the first autonomous navigation of a robotic catheter inside a beating heart using a novel haptic vision sensing modality [66]. The researchers developed a robotic catheter system that uses wall-following algorithms inspired by animals to navigate through the blood-filled heart chambers. The catheter’s tip incorporates a miniature camera and LED encased in a clear silicone optical window, which allows it to image and identify cardiac structures when in contact with tissue. Using ML algorithms, the system can distinguish between blood, tissue, and prosthetic valve surfaces. They implemented two control modes: continuous contact for following ventricular walls and intermittent contact for navigating around the aortic valve annulus. In in vivo porcine experiments, they showed that autonomous navigation from the heart apex to paravalvular leaks was successful in 95% of trials (79/83), with a mean time of 39 ± 17 s. This performance rivaled that of an experienced clinician using either teleoperated robotic control (34 ± 29 s) or manual catheter manipulation (31 ± 27 s). The autonomous system positioned the catheter tip with an accuracy of 3.0 ± 2.0 mm relative to leak locations. Notably, the study demonstrated that autonomous navigation could be achieved using only tip-mounted sensing, without reliance on external imaging. However, limitations include the need for more sophisticated modeling and control techniques for clinical translation, as well as an extension to other minimally invasive procedures and vascular access routes.

Karstensen et al. introduce a modular simulation framework called stEVE (simulated EndoVascular Environment) for endovascular interventions, along with three benchmark environments, to enhance comparability in autonomous endovascular navigation research [65]. The framework allows flexible composition of simulated interventions and seamless conversion of intervention data for RL. The authors propose three benchmarks: BasicWireNav (single guidewire navigation in a fixed vessel geometry), ArchVariety (guidewire navigation in varying patient anatomies), and DualDeviceNav (concurrent manipulation of guidewire and catheter). They demonstrate the framework’s applicability through experiments using deep RL-based autonomous navigation. The controllers were trained in simulation and evaluated both in simulation and on physical test benches with camera and fluoroscopy feedback. Results showed high success rates for BasicWireNav (98/100 in simulation, 97/100 on test bench) and ArchVariety (90/100 in simulation, 84/100 on test bench), with successful transfer from simulation to physical setups. DualDeviceNav achieved a moderate success rate of 40/100 in simulation. The study highlights the potential of stEVE for transferring controllers from simulation to real-world scenarios and its ability to lower entry barriers for endovascular assistance systems research. However, the authors acknowledge limitations in the benchmarks, such as not accounting for device selection, intra-interventional device exchanges, or imaging system repositioning.

**Figure 6:**
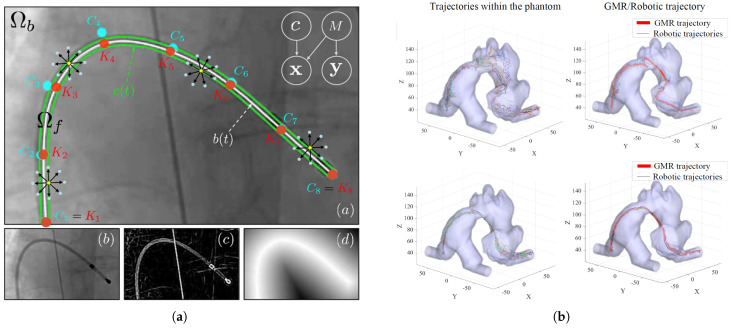

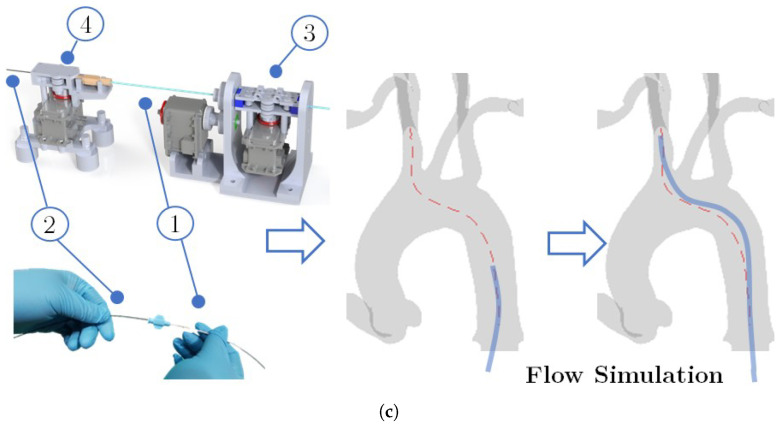
Catheter tracking and autonomous navigation for robot-assisted cardiac interventions. (**a**) B-spline model for catheter tracking [63], (**b**) Catheter tip trajectories inside the phantom model [32], (**c**) Optimized catheter motion trajectory under flow simulation: (1) catheter, (2) guidewire, (3) catheter driver, (4) guidewire driver [64]. Images reproduced with permission.

Jianu et al. introduce CathSim, an open-source simulator for an endovascular intervention designed to facilitate real-time training of ML algorithms for autonomous catheterization [31]. Their main contributions include developing a modular, upgradable, and extendable simulator that balances computational efficiency with realistic physics, and creating an expert navigation network to demonstrate the simulator’s effectiveness in downstream tasks. CathSim incorporates realistic models of the aorta, guidewire, blood simulation, and a robotic follower, all built on the MuJoCo physics engine. The expert navigation network, trained on CathSim, outperformed baseline methods and human surgeons in several metrics, including reduced force exertion, shorter path lengths, and higher success rates in navigating to target arteries. In downstream tasks, the expert navigation network improved imitation learning performance and force prediction accuracy, reducing mean square error from 5.0021 N to 0.0898 N with 100,000 training samples. However, limitations include the use of rigid body assumptions for simplification and potential bias in the expert trajectories generated within the simulator. Wang et al. present a learning-based robotic navigation system for autonomous pulmonary artery catheterization in MRI environments [32]. Their main contribution is the development of a learning-from-demonstration-based Gaussian mixture model (GMM) for robot trajectory optimization, integrated with a 2-DoF MR-compatible interventional robot capable of continuous and simultaneous translation and rotation of a catheter. The system aims to navigate the catheter tip autonomously from the inferior vena cava through the right atrium and right ventricle into the pulmonary artery. The authors collected trajectory data from 50 repetitions of manual and robot-assisted catheterizations in a phantom environment, using a 5-DoF electromagnetic (EM) tracker. They then applied GMM and Gaussian mixture regression to generate optimized trajectories for autonomous catheter navigation. Experimental results showed the robot successfully followed trajectories, with minor deviations in areas requiring greater bending, especially near the right atrium and pulmonary artery. Notably, the robot-assisted procedures were significantly faster than manual handheld procedures, with the autonomous mode being about 15% quicker (approximately 4 s) than the teleoperated mode for catheter advancement. However, limitations include the use of a non-pulsating phantom environment and the need for further testing with patient-specific anatomies. Figure 6 shows the catheter tracking and autonomous navigation approaches used in cardiovascular intervention.

### 2.2. Soft Robotics and Soft Sensors in Robot-Assisted Cardiac Interventions

The recent advancements in soft robotics have led to the development of novel devices [67] and soft sensors [68,69,70] to improve robot-assisted cardiac interventions. Farokhnia et al. introduced a soft robotic inflatable basket catheter designed for improved conformability to the left atrium [71]. Using laser-cut thermoplastic polyurethane to fabricate thin, balloon-like structures, they tested the catheter’s performance in 3D-printed atrial models based on patient CT scans. The optimized designs demonstrated up to 85% sensor coverage across various patient anatomies, significantly outperforming conventional basket catheters. When compared to traditional catheters, soft robotic catheters offer several distinct advantages. First, they provide enhanced conformability to complex anatomical structures, enabling better wall contact in irregular chambers like the left atrium. Second, they demonstrate superior maneuverability in confined spaces due to their compliant materials and distributed actuation mechanisms, resulting in reduced risk of tissue trauma. Third, soft robotic catheters typically achieve higher DoF with simplified actuation methods, allowing for more complex movements with less mechanical complexity. Additionally, their inherent compliance offers improved safety margins during procedures, as they can passively adapt to anatomical boundaries.

Despite these advantages, several challenges must be addressed before the widespread clinical adoption of soft robotic catheters. Biocompatibility and sterilization concerns remain significant, as many soft materials and embedded sensors have not been fully validated for long-term clinical use. Manufacturing scalability presents another challenge, as current fabrication methods often involve complex, multi-step processes that are difficult to standardize for mass production. Control precision and repeatability can be compromised by the inherent non-linear behaviors of soft materials, requiring more sophisticated modeling and control algorithms. Additionally, recent advancements in magnetic continuum robots with multiple stiffness levels have demonstrated significant potential for endovascular interventions, providing enhanced flexibility and control during cardiac procedures [72]. Finally, the integration of sensing modalities within soft structures without compromising their mechanical properties continues to be a technical hurdle that researchers must overcome for effective clinical translation. Addressing these challenges will be crucial for realizing the full potential of soft robotic catheters in routine cardiac interventions. Building upon these fundamental advances in soft catheter design, Mac Murray et al. advanced the field by developing buckled foam actuators with enhanced force characteristics [73,74,75]. They applied this technology to create a patient-specific cardiac compression device, using 3D-printed molds from medical imaging to fabricate a custom-fit foam shell around a porcine heart. The buckled foam actuators achieved approximately double the displacement and 1.4 times the force of uncompressed actuators at the same inflation pressures, with the compression device attaining around 67% of the peak flow rate achieved through manual cardiac massage in ex vivo tests. This approach offers benefits such as rapid fabrication, physician-directed shape modification, and applicability to both adult and pediatric patients; however, the biocompatibility of the materials remains unverified, limiting its clinical readiness.

Recently, Zhang et al. developed bio-inspired soft robots integrating electronic skin with artificial muscles through an in situ solution-based fabrication method for multimodal nanocomposite sensors and actuators [76]. Their soft robots, including the design for a cardiac thera-gripper, were capable of wireless operation and communication. The devices effectively interfaced with biological tissues, enabling real-time sensing and actuation in both ex vivo and in vivo experiments. Nguyen et al. contributed to soft robotic catheter technology by developing a hydraulically actuated catheter capable of bidirectional bending, using a hydraulic filament artificial muscle (HFAM) actuator [77]. They validated analytical models that characterized the relationship between input pressure, HFAM elongation, and the catheter’s bending angle. The catheter demonstrated bidirectional bending, the formation of 3D spiral shapes, and a pulling force of 0.9 N, exceeding typical force requirements for cardiac ablation procedures. Gu et al. introduced magnetic soft-robotic chains (MaSoChains) capable of self-folding into larger assemblies using elastic and magnetic energies, allowing the creation of complex structures at a catheter’s tip [78]. Fabricated through multi-material 3D printing and embedded NdFeB magnets, MaSoChains formed various 2D and 3D geometries, such as an extended-reach catheter tip and a large gripper. This approach expands the potential capabilities of minimally invasive surgical tools; however, using 3D-printed soft materials with time-dependent viscoelastic properties may affect the predictability and scalability of the technology for clinical use. Roshanfar et al. developed a dynamic Cosserat rod-based model for a hybrid-actuated soft robot with stiffness adaptation capabilities, designed for cardiac ablation procedures [38]. They integrated both air pressure and tendon actuation in their model, validating it through experimental testing that compared the robot’s tip position under various pressure and tension conditions. The results showed a low mean absolute error between predicted and measured tip positions, confirming the model’s accuracy in predicting the soft robot’s behavior and ability to adjust stiffness.

**Figure 7:**
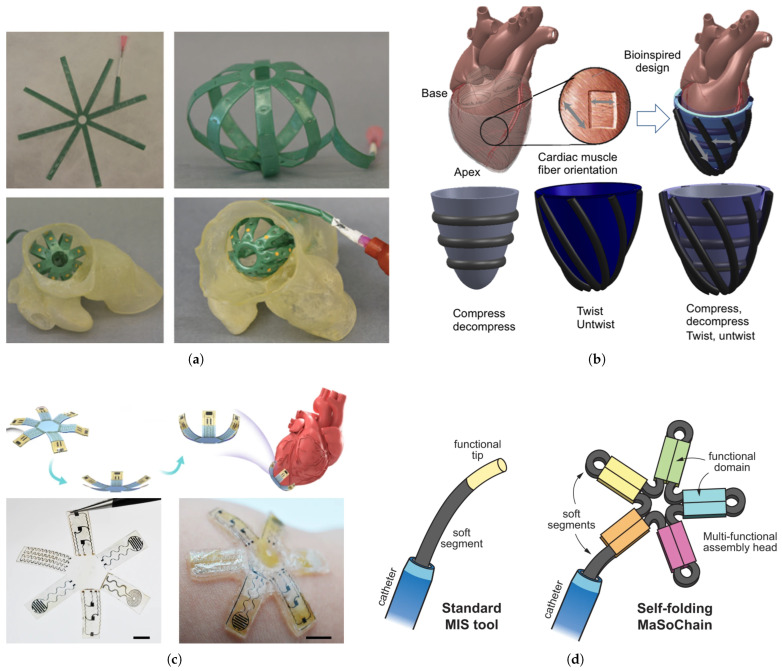
Soft robotics and soft sensors proposed for robot-assisted cardiac interventions. (**a**) Fabrication and conformability of soft inflatable basket [71], (**b**) Conformal soft robotic that can compress and twist [74], (**c**) Bio-inspired sensory robots as minimally invasive smart implants [76], (**d**) Magnetic soft-robotic chains with reconfigurable shapes [78]. Images reproduced with permission.

Kim et al. developed submillimeter-scale soft continuum robots with omnidirectional steering, utilizing ferromagnetic soft materials with programmed magnetic polarities [79]. Their optimization efforts included a theoretical framework and finite element simulations, with a hydrogel skin grown on the robots to reduce friction during navigation. These robots successfully navigated complex vascular phantoms and performed tasks such as steerable laser delivery. Gopesh et al. created a hydraulically actuated soft robotic microcatheter with a steerable tip for the endovascular treatment of cerebral disorders [80]. Fabricated using a multi-step molding process, the device’s performance was assessed through experimental and computational methods, including tests in ex vivo silicone models and in vivo porcine studies. The microcatheter demonstrated guidewire-free navigation, access, and coil deployment in cerebral blood vessels. However, further clinical validation and optimization of the hydraulic actuation system are necessary to fully realize its potential. Figure 7 shows the proposed soft robots and soft sensors designed for use in cardiac interventions.

## 3. Discussion of State-of-the-Art Robotic Technologies and Research Gaps

The advancement of robotic technologies in MIS has led to several innovative approaches addressing various clinical challenges. For instance, Du et al. explored the manipulation skills of interventionists during robot-assisted intravascular PCI by analyzing muscle activity and hand motion data using surface electromyography, EM tracking, and tactile force sensors [81]. Their random forest classification framework, which utilized 19 features to distinguish six types of guidewire movements, achieved a recognition accuracy of 94.11%. The study highlighted correlations between muscle activity and touch force, with higher forces observed in stenotic vascular models compared to smoother pathways. However, limitations such as a small sample size and controlled experimental conditions restrict the generalizability of the results to actual clinical settings.

Kumar et al. contributed to the field by developing miniaturized actuation and sensing units for cardiac ablation catheters [82]. These units employed fiber-reinforced hydraulic actuators and eutectic gallium–indium sensors to enable force generation and sensing capabilities within a range of 0–0.4 N and bandwidths of 1–2 Hz. While the compact design is suitable for MIS, further improvements are required to address biocompatibility concerns and enhance the catheter’s overall design for clinical application. Mao et al. introduced a millimeter-scale magnetic steering continuum robot with follow-the-leader capabilities for transluminal procedures [83]. Utilizing phase transition components to alternately change stiffness, the robot could navigate complex environments independently of tissue interactions and form functional structures in situ. The system demonstrated success in ex vivo and in vivo trials under clinical imaging guidance, offering advantages such as reduced tissue damage and increased mobility. However, its performance may be limited in scenarios where gravity affects its low flexural strength due to a high length-to-diameter ratio. In cardiac ablation, Pittiglio et al. developed a magnetic navigation system featuring a catheter made of spherical permanent magnets, actuated by two cart-mounted rotating permanent magnets. This design achieved tighter bending and higher tip forces compared to conventional magnetic catheters, facilitating the use of smaller actuation magnets while still providing clinically relevant forces [84]. The system’s rotation-based actuation enabled compact integration into clinical settings. Experimental results showed comparable task completion times to manual methods, indicating the potential for smoother and more accurate navigation with adequate training. Rogatinsky et al. presented a multifunctional soft robotic catheter for intracardiac interventions, incorporating a deployable stabilization mechanism and a collapsible soft manipulator to improve outcomes in beating heart surgeries [85]. Their comprehensive evaluation demonstrated the catheter’s ability to maintain consistent contact forces, achieve force outputs suitable for tissue manipulation, and enable tasks like coronary sinus cannulation with efficiency comparable to skilled clinicians.

Lastly, Dreyfus et al. developed a helical magnetic continuum robot designed for improved endovascular access [28]. The robot’s helical outer surface and articulating magnetic tip enhanced navigation through complex vascular structures. In vitro, ex vivo, and in vivo tests validated its capability to navigate tortuous vessels and perform tasks such as fluid injection, with the helical design providing effective propulsion and the articulating tip allowing high steerability even in low magnetic fields. While these state-of-the-art technologies demonstrate significant progress in robotic interventions, several research gaps remain. Common challenges include improving biocompatibility, optimizing device design for clinical use, and overcoming limitations related to robot strength, control, and real-world applicability.

**Figure 8:**
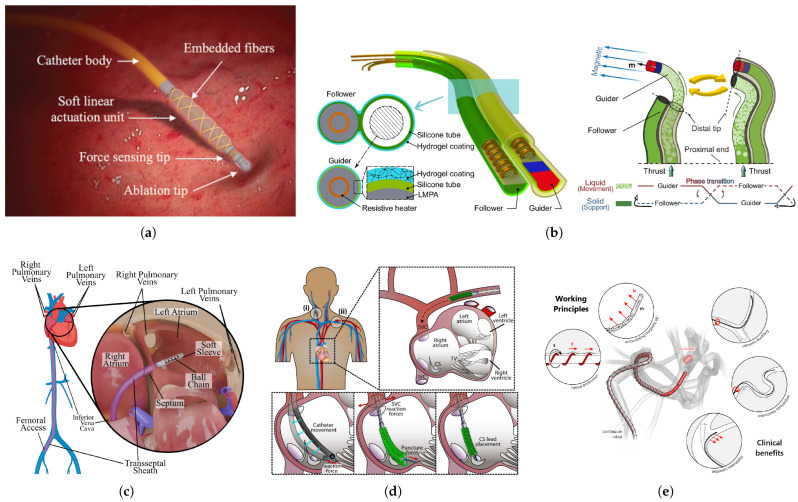
State-of-the-art robotic technologies for cardiac interventions. (**a**) Pneumatically actuated cardiac ablation catheter equipped with integrated soft actuation and sensing unit for real-time force control [82], (**b**) Millimeter-scale magnetic steering continuum robot with programmable shape [83], (**c**) Ball chain magnetic catheter for cardiac arrhythmia treatment [84], (**d**) A multifunctional soft robot for cardiac interventions deploys at the heart’s entrance, maintains contact with moving targets, and produces newton-level forces [85], (**e**) A helical magnetically actuated continuum robot that engages with the vessel wall to convert rotational motion into forward propulsion [28]. Images reproduced with permission.

Another important challenge is developing a molecular-targeted navigation system for robotic interventions. While most approaches for ablation therapy or drug delivery are mapping anatomical or electrical targets, there have been limited technologies for molecular mapping in the cardiovascular system. Quantitative nuclear imaging using single photon emission CT and positron emission tomography, and novel radiotracers targeting cardiac inflammation or fibrosis (e.g., detection of matrix metalloproteinase, integrins, fibroblast activation protein, etc.), may help guide drug delivery or ablation therapies toward the specific underlying molecular signatures or pathological lesion in the heart [86]. The development of an integrated transcatheter miniature nuclear detector with higher spatial and temporal resolution will help this molecular-targeted robotic intervention. Some groups have even integrated radiotracer detection with real-time structural imaging to guide interventional procedures, and further advancements could focus on miniaturizing imaging-compatible robotic platforms, optimizing image-guided control algorithms, and leveraging AI for real-time segmentation and navigation [87,88,89]. Preclinical and clinical validation will be essential to ensure feasibility and accuracy, ultimately bridging the gap between molecular diagnostics and precision robotic therapy for more targeted and effective treatments. Addressing these gaps will be crucial for advancing robotic systems to achieve broader clinical adoption and better patient outcomes. Figure 8 illustrates the state-of-the-art robotic technology platforms proposed for cardiovascular interventions.

## 4. Conclusions and Future Directions

This comprehensive review has explored the cutting-edge technologies shaping the next generation of cardiac interventions, focusing on advanced robotic systems, soft robotics and sensors, and AI applications for autonomous navigation. The integration of these technologies promises to enhance the precision, safety, and efficacy of cardiovascular procedures. So far, significant progress has been made with the development of remote catheter manipulation platforms that offer improved precision and reduced radiation exposure for operators. These systems have demonstrated the feasibility of robotic assistance in complex cardiac procedures, with some novel approaches achieving submillimeter positioning accuracy and improved navigation through intricate vascular structures. Also, soft robotics and sensors have emerged as a promising field for cardiac interventions. Innovations in soft robotic catheters, including inflatable designs and hydraulically actuated systems, have shown potential for improved conformability to cardiac structures and enhanced maneuverability. The integration of advanced sensing technologies has also enabled more precise control and haptic feedback, bringing us closer to replicating the tactile sensations experienced in manual procedures.

On the other hand, AI and autonomous navigation represent another frontier in cardiac interventions. ML techniques have been successfully applied to tasks such as catheter tracking, trajectory optimization, and autonomous navigation in simulated environments. Some systems have demonstrated the potential for reducing the cognitive burden on clinicians during complex procedures, paving the way for more automated and precise interventions. Despite these advancements, several challenges remain to be addressed in future research. Improving the real-time integration of multimodal imaging data is crucial for enhancing intra-operative navigation. This involves developing algorithms for the seamless fusion of different imaging modalities and investigating novel visualization techniques to present complex 3D anatomical information more intuitively to operators. Also, enhancing haptic feedback systems remains a key area for improvement. Future research should focus on developing and refining haptic interfaces to provide more realistic and informative tactile sensations to operators. The integration of advanced sensing technologies with haptic feedback systems could significantly improve the operator’s perception of tissue interaction, leading to safer and more effective procedures.

Validating these advanced technologies in clinical settings is a critical step towards their widespread adoption. Larger-scale clinical trials are needed to validate the efficacy and safety of advanced robotic systems, soft robotic devices, and AI-assisted navigation in diverse patient populations. Developing standardized protocols for evaluating and comparing the performance of different robotic and autonomous systems will be essential for advancing the field. Addressing ethical and regulatory challenges is paramount as these technologies become more autonomous. Frameworks for ensuring appropriate levels of human oversight and intervention in increasingly autonomous cardiovascular procedures need to be developed. Collaboration with regulatory bodies will be crucial for establishing guidelines for the safe implementation and validation of AI-assisted and autonomous robotic systems in clinical practice. By pursuing these avenues of research and development, the field of cardiovascular interventions stands poised to enter a new era of precision, safety, and efficacy. To translate these advancements into clinical practice, several key technical milestones must be met. First, real-time multimodal imaging fusion algorithms must be further refined to enhance accuracy and computational efficiency in robotic navigation. Second, the integration of AI-driven autonomous control systems requires rigorous validation through large-scale preclinical and clinical studies. Third, the development of biocompatible, miniaturized soft robotic actuators with improved durability and responsiveness is essential for their adoption in interventional procedures. Lastly, regulatory frameworks and safety validation protocols must evolve to accommodate the increasing levels of autonomy in robotic-assisted interventions. Addressing these milestones will be crucial in transforming these proposed advancements into widely adopted clinical solutions. The synergistic integration of advanced robotics and soft materials (as the body of the system) and AI (as the brain of the system) has the potential to revolutionize the treatment of cardiovascular disease, ultimately improving outcomes and quality of life for patients worldwide.

## Data Availability

No new data were created or analyzed in this study.

## Abbreviations

The following abbreviations are used in this manuscript:

AF	Atrial Fibrillation
AI	Artificial Intelligence
CAD	Coronary Artery Disease
CT	Computed Tomography
DoF	Degree of Freedom
EM	Electromagnetic
FDA	Food and Drug Administration
FBG	Fiber Bragg Grating
GMM	Gaussian Mixture Model
HFAM	Hydraulic Filament Artificial Muscle
ICE	Intracardiac Echocardiography
ML	Machine Learning
MRI	Magnetic Resonance Imaging
MIS	Minimally Invasive Surgery
MRE	Magnetorheological Elastomer
NCDR	National Cardiovascular Data Registry
NN	Neural Network
PCI	Percutaneous Coronary Intervention
PI	Peripheral Interventions
PVI	Peripheral Vascular Intervention
RCT	Randomized Controlled Trial
RL	Reinforcement Learning
TEE	Transesophageal Echocardiogram
XR	Extended Reality
